# Motor Imagery and Action Observation as Appropriate Strategies for Home-Based Rehabilitation: A Mini-Review Focusing on Improving Physical Function in Orthopedic Patients

**DOI:** 10.3389/fpsyg.2022.826476

**Published:** 2022-03-03

**Authors:** Armin H. Paravlic

**Affiliations:** ^1^Faculty of Sport, Institute of Kinesiology, University of Ljubljana, Ljubljana, Slovenia; ^2^Science and Research Centre Koper, Institute for Kinesiology Research, Koper, Slovenia

**Keywords:** MI, AO, virtual reality, mirror therapy, rehabilitation, physical function, neuromuscular function, mental simulation

## Abstract

Dynamic stability of the knee and weakness of the extensor muscles are considered to be the most important functional limitations after anterior cruciate ligament (ACL) injury, probably due to changes at the central (cortical and corticospinal) level of motor control rather than at the peripheral level. Despite general technological advances, fewer contraindicative surgical procedures, and extensive postoperative rehabilitation, up to 65% of patients fail to return to their preinjury level of sports, and only half were able to return to competitive sport. Later, it becomes clear that current rehabilitation after knee surgery is not sufficient to address the functional limitations after ACL reconstruction even years after surgery. Therefore, new therapeutic tools targeting the central neural system, i.e., the higher centers of motor control, should be investigated and integrated into current rehabilitation practice. To improve motor performance when overt movement cannot be fully performed (e.g., due to pain, impaired motor control, and/or joint immobilization), several techniques have been developed to increase physical and mental activation without the need to perform overt movements. Among the most popular cognitive techniques used to increase physical performance are motor imagery and action observation practices. This review, which examines the available evidence, presents the underlying mechanisms of the efficacy of cognitive interventions and provides guidelines for their use at home.

## Brief Overview of Neurostructural Consequences of ACL Injury and Subsequent Reconstructive Surgery

The knee ligaments injuries are common in pivoting sports (Anderson et al., 2019; Tabben et al., 2020). The high axial and torsional forces applied to the knee joint during sports-specific actions such as sudden change of direction, rapid accelerations and decelerations, along with joint loading occurring following jumping activities represents the main sports-related risk factors for non-contact ligaments injuries (Laible and Sherman, 2014). Serious injuries, such as anterior cruciate ligament (ACL) injury require surgery to restore normal knee function. In elite athletes, this represents a great burden, since it is not uncommon that athletes must be out of the game for a whole season in which injury occurred (Erickson et al., 2013). Thus, return to play might last from 8 months to more than a year, depending on sport and level of play (Bauer et al., 2014; Lindanger et al., 2019; Ekstrand et al., 2020). Long-term effects from such knee injuries alter knee kinematics (Woo et al., 2006; Gardinier et al., 2013) and the joint loading that eventually could result in early-onset of posttraumatic osteoarthritis (Von Porat et al., 2004; Kessler et al., 2008; Friel and Chu, 2013). Despite technological advances in general, less contraindicative surgery procedures (Condello et al., 2019) and extensive postoperative rehabilitation practices (Cavanaugh and Powers, 2017; Andrade et al., 2020), up to 65% of patients fail to return to their pre-injury level of sports and only half were able to return to competitive sport (Ardern et al., 2014). The vast amount of studies were conducted to investigate patients’ functional outcomes after ACL reconstructive (ACLr) surgery (Keays et al., 2007; Eitzen et al., 2009; Abrams et al., 2014). Knowing that motor control/learning strategies rely on integration of afferent information, perceptive functions and efferent commands sent to the periphery, altered afferent input may further induce changes in motor programming and consequently in motor response (Valeriani et al., 1996; Kapreli and Athanasopoulos, 2006). Hence, knee dynamic stability and extensors weakness were reported to be main functional limitations following ACL injury, which is believed to be mostly driven by alterations on the central (cortical and cortico-spinal) level of motor control, rather than the peripheral one (i.e., muscular level) (Morita et al., 2013; Needle et al., 2017; Zarzycki et al., 2020). Besides, strength deficit was shown to be the strongest preoperative predictor of physical function following ACLr (Eitzen et al., 2009). In the first post-operative month, quadriceps muscle strength may reduce by 67% (Kobayashi et al., 2004), and may persist for several years (Keays et al., 2007). Current rehabilitation practice of major knee surgeries consists of a rather conventional approach to exercise that mechanically stresses the musculoskeletal system. Such exercise regimes have incorporated joint mobility exercises aimed in improving range of motion, gait re-education, weight-bearing exercise, training of neuromuscular function and proprioception, as well as strength and endurance exercises, whereas both voluntary and electrically elicited action are used (Kittelson et al., 2013; Andrade et al., 2020). However, given all the above, current post-knee surgery rehabilitation seems to be insufficient to address functional limitations following ACLr even years after surgery (Hunnicutt et al., 2020; Johnston et al., 2020). Therefore, new therapeutic tools targeting central neural system that is higher centers of motor control should be examined and implemented in current rehabilitation practice (Paravlic et al., 2019a).

## Neurostructural Correlates of Strength Decrease in Healthy and Diseased Subjects With Emphasizes on ACLr Patients

It is well known that strength is governed by both neural and structural factors (Gabriel et al., 2006; Folland and Williams, 2007), and it holds through the whole life-span. To construct comprehensive rehabilitation practice and prescribe it with greater certainty, it is imperative to understand the clinical value of skeletal muscle strength and the underlying mechanism of strength weaknesses caused by surgery. For different population in question, it has been suggested that muscle strength deficiency, in particular, represents a significant predictor of future disease (Leong et al., 2015), increased risk of injury (Ribeiro-Alvares et al., 2018), worse rehabilitation outcomes (Mizner et al., 2005a; Brown et al., 2009), and increased all-cause mortality (Brown et al., 2016). Therefore, it represents a fundamental factor to successful and efficient performance execution of many activities of daily living (Ochi et al., 2015; Wang et al., 2020).

While an age-associated reduction in physical function might be partly explained by the aging process itself (Rogers and Evans, 1993), other life circumstances, such as prolonged periods of muscle disuse due to habitual inactivity, illness, immobilization, hospitalization, and/or surgery may underpin rapid deterioration of physical capacities. Research has shown that prolonged physical inactivity has marked a negative impact on both the skeletal muscle structure (Pišot et al., 2008) and function (Pišot et al., 2016). For example, Pišot et al., 2016 reported a decrease of 8.3, 13.2, and 12.3% in quadriceps muscle volume, strength and power, respectively, following 2 weeks of bed rest. These results are in agreement with those reported elsewhere (de Boer et al., 2008; Pišot et al., 2008), and are shown to aggravate with the time of exposure (Pišot et al., 2008).

In periods of hospitalization, following injury (Hortobagyi et al., 2000) or orthopedic surgery, such as ACLr (Palmieri-Smith et al., 2008) and total knee arthroplasty (Paravlic et al., 2019a), the cross-sectional area and strength of the quadriceps may decrease by 10 and 69%, respectively, in early postoperative days. Accordingly, several reasons such as knee pain, joint injury (caused by primary pathology and surgical trauma), use of a tourniquet during surgery and muscle atrophy due disuse may aggravate quadriceps weakness in this early period following a surgery (Brown et al., 2009). However, in last decade a vast amount of studies showing that centrally driven factors are the most prominent indicators of strength loss following a knee surgery (Mizner et al., 2005b; Morita et al., 2013; Needle et al., 2017). For example, Morita et al. (2013), reported the reduction of muscle force by 50 and 37.5% one, and 2 weeks after the unicompartmental knee arthroplasty (Morita et al., 2013). Besides, the active brain region of the sensorimotor leg area narrowed (Morita et al., 2013), whereas the pain severity in the assessed knee 2 weeks postoperatively remained unchanged, suggesting that early postoperative muscle weakness was mostly influenced by the supraspinal pathways (Morita et al., 2013; Needle et al., 2017). Moreover, failure of voluntary muscle activation (VMA) and muscle atrophy together can explain approximately 85% of the quadriceps strength loss, of which the relative contribution of VMA was nearly twice as great as the relative contribution of muscle atrophy to the observed strength decrease at 1-month post-surgery (Mizner et al., 2005b). Muscle inhibition in quadriceps persists for years, and it is often observed bilaterally (Palmieri-Smith et al., 2008; Paravlic et al., 2019a). Indeed, VMA is one of the most investigated proxies of central factors used to assess muscle inhibition related to strength loss following knee surgery (Paravlic et al., 2019a). It represents a major factor in the reduction of maximal force output of the muscle, given a patient’s inability to recruit all of the muscle’s motor units, or a failure to attain the maximal discharge rate from the recruited motor units (Mizner et al., 2005b; Morita et al., 2013). Nowadays, the new technology enables the scientific community to investigate central factors of motor control by more direct type of measurements such as functional magnetic resonance imaging, transcranial magnetic stimulation (TMS) and electroencephalography. The most recent findings (Zarzycki et al., 2021) support the theory that quadriceps dysfunction after ACLr is driven by central factors of motor control. By using TMS (Zarzycki et al., 2021), authors found that short-interval intracortical inhibition (SICI) and intracortical facilitation differ between ACLr patients and healthy controls, whereas SICI showed to be significantly related to quadriceps weakness (Zarzycki et al., 2021). Besides, in another study (Zarzycki et al., 2020) aimed to investigate corticospinal and spinal-reflex excitability in different postoperative time points following ACLr, Zarzycki and co-workers found that resting motor threshold was higher in ACLr group in the non-operated knee and that higher motor-related cortical potential (MRCP) was observed in the operated knee. In contrary, the healthy control group did not show any inter-limb differences in neither time points (for up to 6 months post-surgery) (Zarzycki et al., 2020). These data suggest that ACLr patients had altered corticospinal excitability that did not change for as long as 6 months post-surgery, i.e., a time point when they returned to running activities (Zarzycki et al., 2020). Despite quadriceps strengthening is the main focus of post ACLr rehabilitation programs (Cavanaugh and Powers, 2017; Andrade et al., 2020), strength deficits often continue to exceed pre-operative levels even years following a surgery (Palmieri-Smith et al., 2008). Later imply that current rehabilitation practice needs to be revisited, whereas new and innovative therapeutic tools targeting central neural system i.e., higher centers of motor control should be examined and implemented (Rush et al., 2021). Such strategies were recognized through cognitive practice that has been able to improve the physical function of both symptomatic (Marusic et al., 2018; Paravlic et al., 2020a) and asymptomatic population (Paravlic et al., 2018). Cognitive interventions are often employed by medical staff for restoring a physical function of neurological patients (Abbruzzese et al., 2015; García Carrasco and Aboitiz Cantalapiedra, 2016). Because the exercise requires no special prerequisites and has proven effective in both learning and relearning simple and complex tasks, it is becoming increasingly popular with other populations, from elite athletes to professionals, and is being used more frequently in rehabilitation practise for orthopedic patients.

## Motor Imagery and Action Observation—Evidence-Based Strategies Used to Improve Functional Performance in Orthopedic Patients

To improve motor performance when overt movement cannot be executed to full potential (e.g., due to presence of pain, impaired motor control and/or joint immobilization), several techniques have been designed to increase physical and mental activation without execution of overt movement (Paravlic et al., 2018; Zhang et al., 2018). These mental simulation practice (MSP) techniques have been proven as beneficial tools for strength improvement among different populations (Marusic et al., 2018; Paravlic et al., 2018, 2020a). Among the most popular MSP techniques used to enhance physical performance, the literature highlights MI and action observation (AO) practices (Mulder et al., 2005).

### Motor Imagery

MI practice can be defined as the mental simulation of a specific muscle action without any corresponding motor output (Jeannerod, 1994). Hence, it does not require actual physical movement execution (PME) as a primary tool, making it suitable for various rehabilitation settings. In general, the effectiveness of MI practice relies on functional equivalence principle, which is based on the theory that imagery enhances performance due to the similar neurophysiological processes underlying both imagery and actual movement, and has found its support elsewhere (Jeannerod, 1995; Martin et al., 1999). More precisely, during both motor execution and MI tasks, acute differences have been found in the supplementary motor area, the premotor cortex and the primary motor cortex during imagined or executed movement compared to resting conditions (Guillot et al., 2012). This suggested that imagining the motor task and its actual execution do share similar neural patterns. MI has different modalities such as visual and kinesthetic. Visual MI can be experienced from two distinct perspectives, a first-person perspective (i.e., internal MI) and/or a third-person perspective (i.e., external MI), while kinesthetic MI incorporates including all the somatosensory information normally produced when performing PME (e.g., the sensation of muscle tension, increased blood flow to the working muscles, joint position sense etc.) (Ridderinkhof and Brass, 2015). Studies have shown that the effectiveness of MI depends on both the modality used and the perspective, which is directly related to subject’s ability to imagine the required task (Martin et al., 1999; Ridderinkhof and Brass, 2015). For example, Stinear et al. (2006) demonstrated that kinesthetic, but not visual imagery, modulates corticomotor excitability, primarily at the supraspinal level affecting motor-related structures, whereas visual imagery predominantly affects occipital regions and superior parietal lobes (Guillot et al., 2009). Later studies suggest that the different imagery perspectives are mediated through separate neural systems, which contribute differently during processes of motor learning and neurological rehabilitation (Mulder, 2007). Therefore, to enhance physical performance and achieve different types of outcomes, MI practitioners may use these modalities independently or in combination (Ridderinkhof and Brass, 2015; Paravlic et al., 2018, 2020b). In last decade, a growing number of AO or MI-based interventions studies have been adopted for the rehabilitation of patients with stroke (García Carrasco and Aboitiz Cantalapiedra, 2016), or Parkinson’s Disease (Buccino et al., 2011), and also in orthopedic patients following injury and/or surgery (Marusic et al., 2018; Paravlic et al., 2020a) of which some reported equivocal results (Paravlic et al., 2020b). One of the first studies conducted within ACLr patients population used a combination of relaxation and guided imagery practice constructed to affect patients knee strength, reinjury anxiety and pain (Cupal and Brewer, 2001). Authors found that 10 sessions over 6 months had a beneficial effect on all measures assessed when compared to the control group. Despite positive effects were found, the latter study did not investigate any possible mechanisms underlying MI effectiveness. Hence, two more recent, exploratory studies (Lebon et al., 2012; Maddison et al., 2012) showed promising results on underlying mechanisms of MI practice in this population. Maddison and co-workers (Maddison et al., 2012), found that neurobiological factors such as noradrenaline and dopamine levels were consistently lower in the MI compared to control group across all time-points (2, 6 and 12 weeks post-surgery). However, no differences were found in muscle strength between groups (Maddison et al., 2012). Thus, providing preliminary evidence that MI practice was associated with a reduction in stress levels, that might mediate a post-surgery healing. Non-significant results in strength, may be prescribed to different imagery modalities and tasks used, whereas MI of strength related task was not the primary focus of the prescribed treatment (Maddison et al., 2012). Finally, Lebon and co-workers found that 12 sessions of MI elicited greater electromyographic activity of the quadriceps muscle, while no differences in alleviation of pain were observed between groups. On the other hand, Paravlic et al., 2020a showed that MI training when added to routine physical therapy improved both objective and subjective measures of patients’ physical function at 1 month following total knee arthroplasty. Briefly, they found that MI task consisted of maximal voluntary isometric contraction (MViC) task: 15 min/day; five times/week for 4 weeks in duration, ameliorate strength, VMA, gait speed, along with functional performance task measures assessed by timed-up to go and sit/to stand tests, respectively. Thus, knowledge gained through latter study, might have a great potential to be effectively translated to rehabilitation of ACLr patient’s population.

### Action Observation

Unlike MI, AO practice requires the subject to observe a video or direct actions performed by an operator (Marusic et al., 2018). On the other hand, AO, similarly to MI and PME, shares common neurological basis attributed to the mirror neuron system (Cattaneo and Rizzolatti, 2009; Van Gog et al., 2009). In humans, the areas active during MI, PME and AO are found in the frontal and parietal lobes, whereas the activation of the mirror neuron system is related to the experience the subject has with the observed and/or imagined action (Calvo-Merino et al., 2005; Olsson and Nyberg, 2010). A parieto-frontal network with a proportion of mirror neurons has been recognized as the neural substrate that transforms a visual information into cortical areas and enables motor execution (so-called visuomotor transformation) (Rizzolatti and Sinigaglia, 2010). Subsequently, various AO modalities have been developed in recent years, of which watching a real video-recorded action and PME with mirror visual feedback (MVF) are the most commonly used in rehabilitation practice (Sarasso et al., 2015; Marusic and Grosprêtre, 2018; Zhang et al., 2018), while virtual reality (VR) training is becoming increasingly popular. In general, AO has been shown to be an effective strategy for treatment of stroke (García Carrasco and Aboitiz Cantalapiedra, 2016), Parkinson’s disease (Buccino et al., 2011), cerebral palsy (Sarasso et al., 2015), and orthopedic patients following total knee arthroplasty (Villafañe et al., 2017). Villafañe et al. (2017) investigated whether self-administered AO therapy, compared to routine physical therapy, can enhance the effects of inpatient rehabilitation practice following primary total knee arthroplasty. The authors found greater improvements in the AO group in active knee range of motion, while finding no significant differences between groups in Short Form-36, Lequesne, Barthel Index, and Tinetti score. A recent study by Marusic et al. (2018) reported promising results using the combination of AO with MI as an adjunct therapy to routine rehabilitation practice after total hip arthroplasty (Marusic et al., 2018). Marusic et al. (2018) found that the experimental group performed significantly better on the timed-up-to-go test, four-step square test, and dual-task performance during fast walking compared to the control group. This suggests that the combination of AO and MI can be an effective adjunct tool to improve rehabilitation outcomes in the orthopedic population when considering objective measures of physical function.

On the other hand MVF therapy has been effectively used in neurorehabilitation of stroke patients and is thought to promote neuroplasticity in brain regions involved in sensory normalization and motor recovery (Reynolds et al., 2015; Zhang et al., 2018). MVF involves positioning a mirror in the midsagittal plane to replace the actual image of the affected (i.e., injured) limb with the mirror reflection of the unaffected side while performing PME of the required task. Mostly, it is used for therapeutic purposes for upper limb motor function. However, a recent systematic review and meta-analysis examined the efficacy of MVF for improving lower limb function in stroke survivors (Louie et al., 2019). The authors found significant results in favor of MVF therapy compared to control intervention for gait speed, mobility, and motor recovery (Louie et al., 2019). Although there is no study to date that has investigated the efficacy of MVF in orthopedic patients, later results suggest that MVF therapy may be a promising tool for improving physical function after lower limb immobilization and/or surgery. Therefore, experimental studies in this field are warranted.

With a rapid technological development, the technologies used in the gaming have become more affordable and thus, increasingly popular for both the entertainment and research purposes. In the last two decades, the virtual reality (VR) training was introduced as a type of MSP that is supposed to be a more engaging, motivating, and more stimulating AO concept for different user groups compared to the traditional type of training (Donath et al., 2016). From a technological point of view, VR training would compensate for the practical shortcomings of MI and AO training and contribute significantly to the improvement of motor performance and the quality of external stimuli, which are often not present in MI and traditional AO forms of training. Recent evidence suggests that VR-based rehabilitation has the potential to improve balance and gait outcomes in neurological patients (Parkinson’s disease, multiple sclerosis, acute and chronic stroke, etc.) (Leong et al., 2015; Andrade et al., 2020). The underlying mechanism of VR efficiency has been described mainly through motor learning mechanisms (Freeman et al., 2017). The nature of engagement on VR allows training and/or rehabilitation exercises to feel similar to physically performed actions (Proffitt and Lange, 2015). With the immersive nature of VR, which includes three-dimensional environments displayed to the viewer through head-mounted display, afferent sensory stimuli could be further augmented, potentially improving perception and motor reprogramming (You et al., 2005; Cho and Lee, 2013; Singh et al., 2013). Knowing that the benefits of cognitive intervention strategies could be augmented when the principal components of the functional equivalence model are met (Wright and Smith, 2009), we believe that VR strategies could improve the existing disadvantages of MI and AO based interventions, which remains to be explored.

### Combination of Motor Imagery and Action Observation

Traditionally, AO and MI have been considered as independent intervention methods, however, researchers have begun to consider the possibility of combining those two techniques into a single intervention strategy to facilitate (re-)learning and rehabilitation of motor skills to a larger extent (Paravlic et al., 2020a). Previous work has revealed that both techniques when used independently, activate similar brain regions as actual motor execution and increases corticospinal excitability (Wright et al., 2014), while its combination might elicit greater activation of corticomotor regions than AO (Nedelko et al., 2012) or MI alone (Wright et al., 2014). Positive changes following combined MI and AO were explained throughout greater facilitation of corticospinal excitability, further contributing to greater neural impulse output to agonist’s muscles, and finally increasing muscular activity. Consequently, this might lead to better synchronization of the fibers and inhibition at the level of antagonist muscle activation, hence improving force output (Paravlic et al., 2018). Thus, a combination of AO and MI may have important implications for neurorehabilitation and motor performance enhancement (Marusic et al., 2018; Paravlic et al., 2020b).

Examination of studies investigating MSP effectiveness in patients physical function rehabilitation following major orthopedic surgeries reveals a high heterogeneity among types and duration of cognitive interventions, frequency of exposure, follow-up periods, and the variety of physical function assessment tools used. Our research group (Paravlic et al., 2020b) has conducted and published the most recent systematic review in the field aimed to investigate cognitive interventions effectiveness by rigor methodology implemented (Paravlic et al., 2020b). Results showed that cognitive intervention when added to routine physical therapy has multiple positive effects on measures of physical function recovery in patients after major hip and/or knee surgery, in comparison to routine physical therapy alone (Paravlic et al., 2020b). Besides, it was found that MI intervention has the most substantial effect when compared to AO or their combination (MI + AO), supporting recent experimental findings (Cuenca-Martínez et al., 2018). A later study (Cuenca-Martínez et al., 2018) concluded that AO of a simple motor task prior MI did not facilitate subsequent imagination to a greater extent than MI alone. However, the aforementioned results could not be fully generalized to the symptomatic population suffering from pain and living with some type of functional disability, as the authors only recruited healthy adults. Further investigation into the differences in neurophysiological responses and treatment efficacy between various combinations of MI and AO in the symptomatic population performing complex movements is warranted.

## Recommendations for MI and AO Rehabilitation Practice at Home-Based Setting

There is a need to propose guidelines for the MSP intervention to facilitate its effectiveness in the home setting. Paravlic et al. (2018) showed that several motor imaging variables were associated with improved strength: a training duration of 4 weeks, a training frequency of three sessions per week, a training volume of two to three sets, 25 repetitions per set, and a single session duration of 15 min (Table 1). Since, the efficacy of MSP depends on the patient representation of a particular motor task, it is highly recommended to assess the patient’s ability to imagine the desired task at the beginning of the MSP intervention. This can be done with the Motor Imagery Questionnaire (MIQ-3) (Paravlić et al., 2018) when other, more comprehensive tools to assess the central and/or autonomic nervous system are not available. Although previous literature has shown MI + AO does not promote autonomic nervous response to a greater extent than MI alone (Cuenca-Martínez et al., 2018), there is evidence that both MI and AO can improve the mental representational structure and the accuracy of a golf putting task during the early learning phase in novice golfers (Kim et al., 2017). Moreover, the authors showed that perceptual-cognitive changes were associated with the change in skill performance only after AO (Kim et al., 2017). Therefore, patients who cannot imagine and/or perform the required movement well could start with the simple AO exercise by watching the videotape of the actual performance of the task, to facilitate motor learning and acquire a better representation of the motor task. To date, no study has examined the difference between different combinations of AO and MI sequence order. Therefore, no definite conclusion can be drawn on this issue. However, AO can be used in both the early and later stages of the rehabilitation protocol. The goal is to facilitate motor learning when more complex tasks are included in the rehabilitation programme. In addition, it would be advisable to give the patient instructions in the form of audio tapes to follow. This will make the entire MSP process easier to understand and follow (Paravlic et al., 2019b, 2020a).

**TABLE 1:** Training variables with largest mean effect on maximal muscle strength.

Training variables	Motor imagery vs no-exercise controls	
	Highest value	Effect size
Training period (weeks)	4	0.88
Training frequency (per week)	3	1.22
Number of sets (per training)	2–3	0.90
Number of repetitions (per set)	25	1.18
Number of repetitions (per one session)	50	1.18
Number of repetitions (per study)	1,000	1.18
Training intensity (% of 1RM or MVC)	100	0.92
Time under tension (s)^a^	5	1.05
Rest in between sets (s)	20	1.20
Rest in between repetitions (s)	5	1.37
Total training duration per study (min)	300	1.07
Total training duration per week (min)	60–80	0.99
Duration of one training session (min)	15	1.04

*The content of this table is based on individual training variables with no respect for interaction between training variables.*

*1RM one-repetition maximum, MVC maximum voluntary contraction.*

*^a^Time under tension was calculated only for MViC contraction (100% intensity).*

**FIGURE 1:**
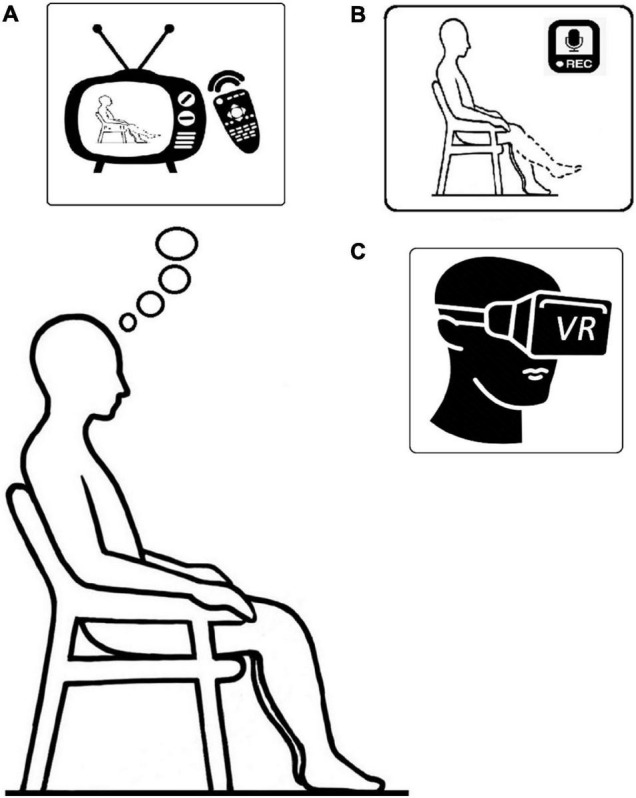
Illustration of: **(A)** action observation; **(B)** motor imagery; and **(C)** virtual reality, training setup respectively.

An example: imagine you are at the gym, sitting at the leg curl machine (while actually sitting in the chair) (Figure 1). Relax, assume a normal posture, and imagine that you are performing a knee extension exercise by isometrically contracting your thigh muscles to your perceived maximum (i.e., maximum effort). One repetition should last 5 s, followed by a 5-s rest period between repetitions. In addition, take a 20-s rest after every fifth contraction. We repeat this process five times for a total of 25 contractions. After a countdown from three to one, get ready to begin the first set of the exercise. Three, two, one—START, five, four, three, two, one, RELAX—and repeat the instructions as directed.

If you are following the primary principle of exercise, which is progression, you should practice MSP more frequently and with greater intensity and volume, starting with one to three sets per session and progressing to more sets and more frequent practice. Example: To comply with the basic principles of strength training, i.e., gradual overload and progression, 10 additional attempts should be added in weeks three and four, respectively.

## Future Perspectives and Recommendations for Improvement of Cognitive Based Rehabilitation Strategies

While MI or AO exercise thought to be effective when used either independently or combined, there is great variability in the magnitude of the effect of exercise between individuals in both the short-term and longitudinally designed experimental studies (Lebon et al., 2012; Paravlic et al., 2019b). Observed variability among responders and non-responders to MI training intervention could be attributed to imagery type and perspective used (Stinear et al., 2006) as well as individual differences in the ability to imagine and execute the movement task (Martin et al., 1999; Olsson and Nyberg, 2010). Thus, Stinear et al. (2006) demonstrated that kinesthetic, but not visual imagery, modulates corticomotor excitability, primarily at the supraspinal level affecting motor-related structures, while visual imagery predominantly affects the occipital regions and the superior parietal lobules (Guillot et al., 2009). These results suggest that different imagery perspectives are mediated through separate neural systems, which contribute differently during processes of motor learning and neurological rehabilitation (Mulder, 2007). Besides, previous experience or gaining experience by observing repetitive movements may facilitate motor learning of the specific task (Ito et al., 2020). However, the discrepancies between the different types and perspectives of cognitive practice, as well as the control of the subjects’ actual involvement in the practice, could be overcome by modifying and adapting old strategies such as PME with MVF to the rehabilitation practice of patients with orthopedic lower limb disorders, or by using a new tool such as the VR practice.

This article presents various MSP strategies such as MI and different AO modalities that can be efficiently integrated into rehabilitation practice for orthopedic patients. While MI has been shown to be more efficient in improving physical function in symptomatic populations with limited lower limb function compared to AO or MI + AO, there is a lack of evidence when considering MVF and VR. Therefore, future studies examining differences in neurophysiological responses and treatment efficacy between various combinations of MI and AO, and MVF and VR in orthopedic patients are warranted.

## Author Contributions

The author confirms being the sole contributor of this work and has approved it for publication.

## Conflict of Interest

The author declares that the research was conducted in the absence of any commercial or financial relationships that could be construed as a potential conflict of interest.

## Publisher’s Note

All claims expressed in this article are solely those of the authors and do not necessarily represent those of their affiliated organizations, or those of the publisher, the editors and the reviewers. Any product that may be evaluated in this article, or claim that may be made by its manufacturer, is not guaranteed or endorsed by the publisher.
